# Confronting species aesthetics with ecological functions in coral reef fish

**DOI:** 10.1038/s41598-018-29637-7

**Published:** 2018-08-06

**Authors:** Anne-Sophie Tribot, Quentin Carabeux, Julie Deter, Thomas Claverie, Sébastien Villéger, Nicolas Mouquet

**Affiliations:** 1 MARBEC, Univ Montpellier, CNRS, Ifremer, IRD, Montpellier, France; 2 ISEM, Univ Montpellier, CNRS, IRD, Montpellier, France; 3 Andromède Océanologie, Carnon, France; 4 Centre Universitaire de formation et de recherche de Mayotte, Dembeni, Mayotte

## Abstract

The biodiversity crisis has spurred scientists to assess all facets of biodiversity so that stakeholders can establish protection programs. However, species that are perceived as beautiful receive more attention than less attractive species. This dynamic could have tremendous consequences on people’s willingness to preserve biodiversity. Coral reefs might be particularly affected by this issue as they are key ecosystems that provide many services, such as aesthetic and cultural benefits attracting millions of tourists each year. Here we show the results of an online photographic questionnaire completed by 8,000 participants whereby preferences were assessed for a set of 116 reef fishes. Based on these preferences, we compared the functional richness, i.e. the amount of functional space filled, by groups of fishes based on their perceived attractiveness. We present evidence indicating that the least attractive coral reef fishes have a much higher functional richness than the most attractive species. Our results highlight the extent to which species aesthetic values’ may be disconnected from their ecological values and could be misleading for conservation purposes. There is thus an urgent need to increase the attention of scientists and the general public towards less attractive species to better appreciate and protect the species that crucially support functional diversity in endangered ecosystems.

## Introduction

The human perception of nature is one of the building blocks of conservation policies. However, our individual relationship with biodiversity is strongly biased by our capacity to analyse and interpret natural phenomena as well as by our cultural heritage and social background characteristics^1,2^. A simple and intuitive example of these biases is the tendency of the general public and scientists to take more interest in beautiful and attractive species^3^. For instance, flagship species (aesthetically appealing, and generally with a large body mass^4^) are intended to promote public awareness and to raise funds for conservation programs^5^. However, conservationists have long recognized that flagship species campaigns should be used with caution because they could bias conservation toward a limited range of species^4^. Although commonly accepted, this idea has not yet fully percolated into biological conservation programmes and ecological research agendas^6^. These biases could, however, have profound consequences in the context of the current biodiversity crisis, for which choices must be made in conservation efforts to preserve biological diversity and ecosystem functioning and services.

For instance, a tremendous amount of effort has been invested in studying the relationship between biological diversity and ecosystem functioning (BDEF), and the consensus that species richness positively influences ecosystem functioning^7^ has emerged. However, there is also evidence that all species do not contribute equally to ecosystem functioning and that functional traits, more than species numbers *per se*, are key elements of the BDEF relationship^8^. In this context, any bias in the human perception of nature, and therefore in the willingness to conserve biological diversity, could have profound consequences for conservation and thus the functioning of endangered ecosystems. More generally, aesthetic value is considered a cultural ecosystem service and is acknowledged as a strong driver for conservation^9^. However, aesthetic value has not yet been fully integrated into current attempts to link biodiversity and ecosystem services^10^. There is thus an urgent need to quantify how species aesthetic values are related to their ecological attributes^11^.

This issue particularly concerns taxon with variation in shape and colors patterns sufficiently large to trigger contrasted emotional responses (e.g. birds, fishes, reptiles, amphibians and mammals). Among these, coral reef fishes are potentially concerned as they are a very rich group of up to 8,000 species, including some emblematic species^12^, widely publicized in the media^13^, such as clownfish, as well as many colourful species popular among aquarists^14^. However, beautiful species are not the sole components of coral reefs fish communities, and a lack of attention towards less attractive species may alter human ability to protect them. Coral reefs that are among the most important ecosystems on Earth because their productivity and biological diversity provide many goods and services to humans^13,15^. Coral reefs are also suffering from a dramatic global decline due to anthropogenic-induced stress that exceed their regenerative capacity^15^. Assessing how functional diversity is distributed along a continuum of aesthetic preferences will therefore help to prevent any potential cultural bias in conservation policies and research programs on this endangered biodiversity.

## Results and Discussion

### Aesthetic value of coral reef fishes

**Figure 1:**
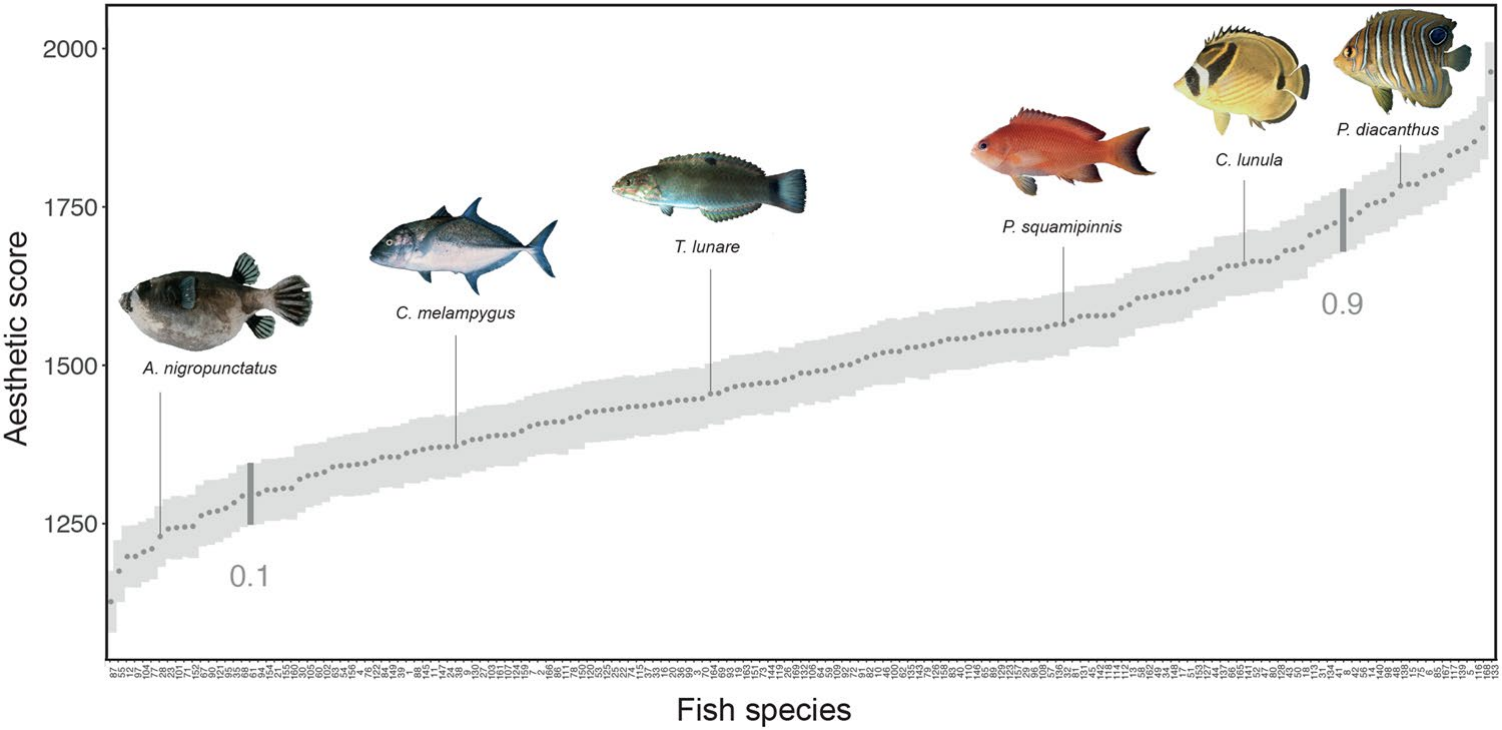
Mean aesthetic scores. Grey points represent the mean aesthetic scores, and shaded areas show standard deviations. The vertical segments highlight the first (0.1) and ninth (0.9) deciles of the distribution of aesthetic scores. Fishes shown left to right are as follows: *Arothron nigropunctatus* (mean aesthetic score = 1231); *Caranx melampygus* (1373); *Thalassoma lunare* female (1456); *Pseudanthias squamipinnis* female (1566); *Chaetodon lunula* (1665); and *Pygoplites diacanthus* juvenile (1758). Photographs: Randall, J. E. from FishBase.org.

To assess the human aesthetic preferences (attractiveness) for coral reef fishes, we selected 169 reef fish photographs depicting 116 dominant fish species from the western Indian Ocean, representing 29 of the 48 most dominant families of coral reef fishes (see Methods ‘Choice of photographs’)^12^. We calculated aesthetic scores for each photo by computing anonymous and online random photographic pair questionnaires to 8,000 participants (see Methods ‘Choice of photographs’, ‘Aesthetic score calculation’ and Supplementary Fig. 1). The photographs were ranked using the Elo algorithm, which is based on pairwise comparisons^16^. We found a normal distribution for the mean aesthetic scores (p-value for Shapiro Test = 0.1759), which ranged from 1128 to 1964 with a mean standard deviation of 48 (+/−1.427) (Fig. 1, see also Methods ‘Aesthetic score calculation’). Overall, we found no significant effect of the social background characteristics of the observers on aesthetic preferences, except for diving experience, which had a marginal impact on aesthetic scores (see Methods ‘Effect of social background characteristics’). Non-divers preferred fishes with the typical shape called compressiform (e.g. Pomacentridae), whereas divers preferred fishes with unusual shapes such as globiform (e.g. Tetraodontidae), anguilliform (e.g. Muraenidae) and sagittiform (e.g. Aulostomidae, Supplementary Fig. 2).

### Functional diversity of more and less attractive fishes

**Figure 2:**
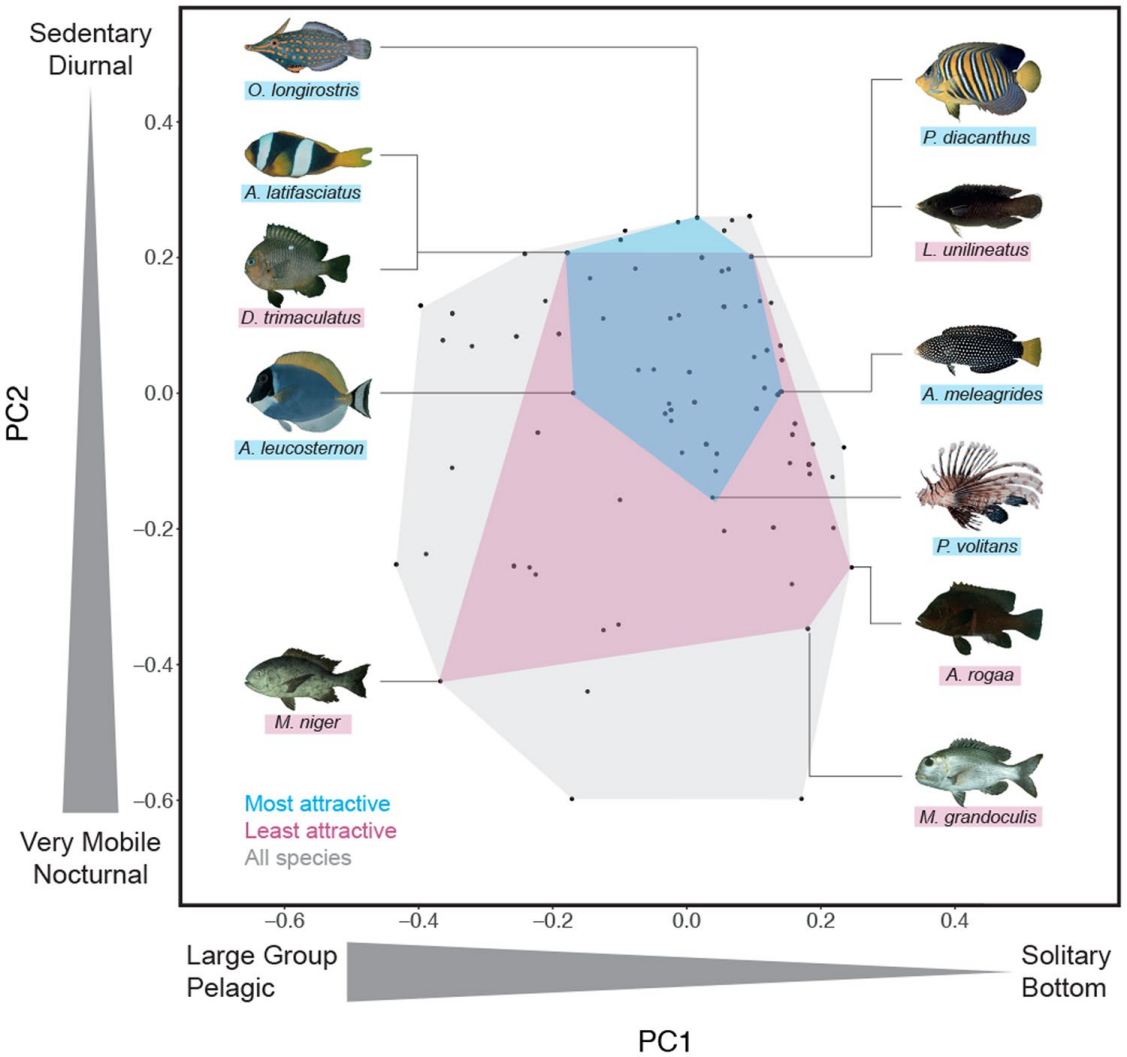
Functional space of the species pool. PC1 and PC2 represent the two first axes of the functional space and vary according to schooling and position for PC1 and mobility and activity for PC2. Each black point represents the position of each species within the functional space. The functional space filled by the first and ninth deciles of the aesthetic score distribution (i.e. the 18 species with the highest and lowest aesthetic scores) are represented by the blue and pink areas, respectively. Fishes shown top to bottom and left to right are as follows: *Oxymonacanthus longirostris*, *Amphiprion latifasciatus*, *Dascyllus trimaculatus*, *Acanthurus leucosternon*, *Macolor niger*, *Pygoplites diacanthus*, *Labrichtys unilineatus* female, *Anampses meleagrides* female, *Pterois volitans*, *Aethaloperca rogaa*, and *Monotaxis grandoculis*. Photographs: Randall, J. E. from FishBase.org.

To highlight how functional traits were distributed among all species, we selected a set of six traits describing complementary facets of fish biology (the GASPAR database): size, mobility, time period of activity, type of grouping, position in the water column, and diet (see Methods ‘Functional space computation’, Supplementary Fig. 3 and^17^). These traits are all linked to the ecology of the species and thus to ecosystem processes such as regulation of food webs and nutrient cycling^17^. To assess functional diversity, i.e. the amount of functional space filled by a set of fish species, we built a multidimensional functional space based on the trait values (see Methods ‘Functional space computation’ and^18^). We found that the most attractive fishes (aesthetic scores in the ninth decile of the distribution, n = 18) filled a much smaller part of the total functional space (20% of the total space) than the least attractive fishes (aesthetic scores in the first decile of the aesthetic scores distribution, n = 18, 40% of the total space). The most attractive fishes were aggregated in the top right of the functional space, corresponding to sedentary, diurnal, living in pairs or small groups and found in the lower part of the water column (Fig. 2). Among the most attractive fishes were the clownfish (*Amphiprion latifasciatus*) and the lionfish (*Pterois volitans*). By contrast, the least attractive fishes were distributed across all the parts of the functional space and therefore represented a greater diversity of functional traits.

**Figure 3:**
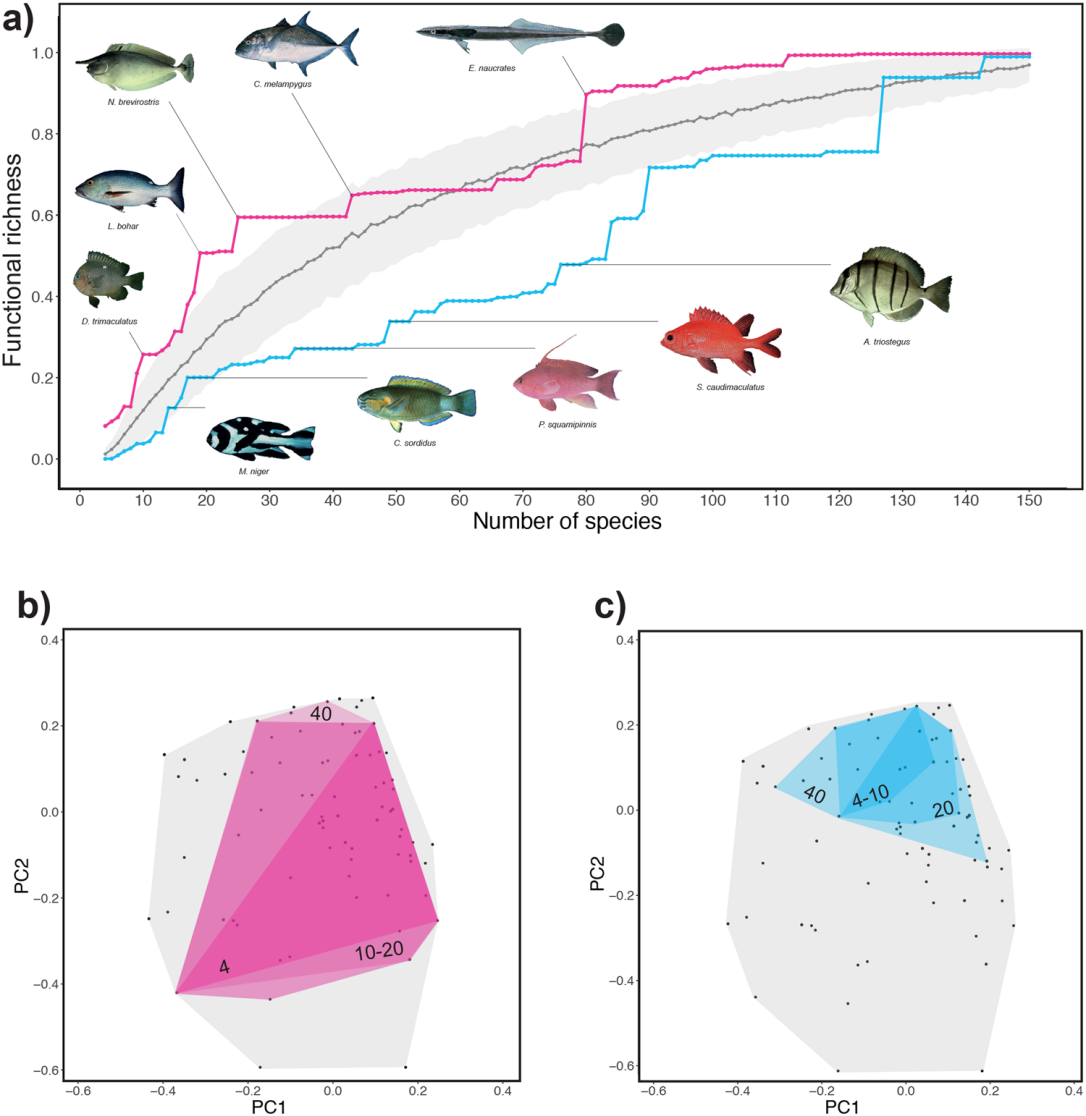
Functional richness of the more and less attractive fishes. (**a**) Functional richness (FRic) of groups of fishes ordered by descending or ascending attractiveness from 4 to 169 fishes. The grey line shows the mean expected FRic for groups of randomly sampled fishes (see Methods ‘Functional richness of more and less attractive species’), and shaded areas represent the standard deviations (more than 1,000 replications). Fishes shown left to right and top to bottom are as follows: *Naso brevirostris*, *Caranx melampygus*, *Echeneis naucrates*, *Lutjanus bohar*, *Dascyllus trimaculatus*, *Macolor niger* juvenile, *Chlorurus sordidus* male, *Pseudanthias squamipinnis* male, *Sargocentron caudimaculatum* and *Acanthurus triostegus*. Photographs: Randall, J. E. from FishBase.org. (**b**) Functional space filled by the 4, 10, 20, 30 and 40 most and (**c**) least attractive fishes.

To test the robustness of this finding, we sampled groups of fishes according to ascending and descending aesthetic scores, starting with the four most and least attractive fishes and then expanding to all fishes, and compared the realized functional richness index, FRic^18^, of each group (Fig. 3a). This index quantifies the amount of the total functional space filled by each group of fishes and was compared to the functional richness of random groups of fishes of same size (see Methods ‘Functional richness of more and less attractive fish’). We found that on average, the least attractive fishes had a functional richness 33% higher than that of the most attractive fishes (Fig. 3a, see also Methods ‘Functional richness of more and less attractive fishes’). The functional space filled by the least attractive fishes spread very rapidly when the number of fishes increased (4, 10, 20, 30 and 40 fish; Fig. 3b,c), whereas the functional space filled by the most beautiful fishes remained small. The 20 least attractive fishes represented a significant proportion (more than 50%) of the functional traits provided by the global pool of fishes.

### Effect of taxonomy on aesthetic value

These results suggest that attractive species represent less functional diversity and thus a smaller range of ecological roles in ecosystems than less attractive species. Generally, the visual attributes that make species - or other objects - attractive are relatively similar: bright colours or the presence of contrasting patterns^19^. Although colour parameters and contrast intensities were not formally measured in this study, we see at a glance that these attributes were not evenly distributed among taxa. For instance, Chaetodontidae, commonly called ‘butterflyfish’, owe their name to their colourful and luminous appearance and are adorned with black bands or circular spots^20^. Unsurprisingly, we found that membership in the family Chaetodontidae (mean aesthetic score = 1678, n = 14) had a significantly greater effect on aesthetic preferences than membership in other families (p-value < 0.001, see Methods ‘Effect of taxonomy on aesthetic scores’ and Supplementary Fig. 4). This bias in visual attractiveness resulted in the indirect functional bias that we found. Translated within into functional terms, this means that based on visual characteristics, people appreciate fishes that are sedentary and diurnal, that live in pairs or small groups and that are found in the lower part of the water column (Fig. 2).

### Aesthetic bottleneck and conservation

Our results highlight the extent to which aesthetic value may be disconnected from the functioning of ecosystems. Attractive visual attributes were found in species that are ecologically close, leading to an ‘aesthetic bottleneck’. More generally, using species’ attractiveness to motivate conservation prioritization (via public support) could lead to overlook a large amount of the essential functional diversity in ecosystems. Communication campaigns based on charismatic species reinforce this ‘aesthetic bottleneck’, and do not promote the general public awareness on the need to conserve functional diversity supported by less attractive species. This aesthetic bias, although measured for some taxa^6,21^, still needs to be measured globally to promote conservation and research efforts for less attractive species. This bias has some evolutionary origins^22^ and is shaped by cultural contexts^23^ that will need to be disentangled to understand its nature. For example in the case of this study, the aesthetic scores reflect primarily preferences of presumably Europeans who are distant from, and therefore largely unfamiliar, with western Indian Ocean reefs.

Understanding this bias will also help to improve the efficiency of conservation policies by including human perception dimension into conservation programs. We also acknowledge that conservation is not always made at a species level, and that to fully measure the consequences of this aesthetic bias, evaluation should be conducted at the community and ecosystem levels. Ultimately, understanding the scaling of this bias will help connect human aesthetic culture with ecological phenomena^24^ and reinforce our social motivation to conserve biological diversity.

Our evaluation of aesthetic value of coral reef fishes was based on individual photos and we acknowledge that some species could have different aesthetic scores when observed in the field. This is particularly true for schooling, for instance when some species - such as *Naso brevirostris* - considered less attractive individually could be judged more attractive if viewed as a shoal. Interesting behaviours such as cleaning stations might also attract attention and change aesthetic value for some species (e.g. cleaning wrasse). Finally, the images used did not allow the observer to take into account the size of the fish, while the size of the species can influence their attractiveness^4^. Future evaluation will thus have to compare individual based aesthetic scores with more “realistic” situations in order to disentangle the aesthetic contribution of species in communities (i.e. by using assemblages of fishes with different levels of diversity, abundances, and sizes). Integrating information on species aesthetic value at the community level will also be necessary to implement conservation actions for coral reef ecosystems. Despite the most attractive coral reef fishes have a much lower functional richness, understanding how they are associated with less attractive species in natural communities will provide valuable information for policy makers. For instance, we found that Chaetodontidae were very attractive, but they are also recognized as bioindicators for coral reef deterioration^25^. This family could thus be a good candidate to serve as umbrella or flagship species.

Evidence of the importance of species diversity on human perception of ecosystems is increasing^26,27^, but no study has yet measured the relationship between species attractiveness, ecosystem functioning and the motivation for conservation. This issue will be crucial in developing operational conservation programmes based on a good understanding of the human perceptions of species and ecosystems. For instance, increasing public knowledge and understanding the ecological roles of species could create a positive aesthetic ecological experience^28,29^ that may even trigger emotional learning feedbacks^30^ that deeply modify our cultural bias. We have shown here that least attractive species that represent an ‘overlooked diversity’ are essential to the functioning of ecosystems. Such species call into question our intimate motivation to conserve biodiversity and spur a better understanding of our emotional connections to nature based on aesthetic perceptions.

## Methods

### Choice of photographs

To assess human aesthetic preferences for coral reefs fishes, we chose 116 common coral reef fish species from the western Indian Ocean, representing 29 of the 48 most dominant families of coral reefs fishes (Supplementary Table 1,^12^). The photographs were collected from FishBase^31,32^. All photos were standardized to 400 × 600 mm at 150 dpi, the size of each of each fish has been standardized, and a black background was added. All Different photos were used for species presenting a differentiation between males and females (e.g. *Thalassoma purpureum*), between adults and juveniles (e.g. *Plectorhinchus vittatus*) and colour polymorphism (e.g. *Arothron meleagris* was represented nine times). This process resulted in a total set of 169 photographs. Adults and juveniles of the same species were treated as effectively different species in analyses.

### Aesthetic score calculation

We used an anonymous online photographic questionnaire that was available to the general public on a dedicated website between March and June 2016 (8,000 answers were collected). For each participant, the questionnaire consisted of a random sampling without replacement of 20 pairs (40 random photos in total) among 169 standardized photos of individual fishes. For each pair (hereafter ‘match’), the participants had to choose the photo they felt to be the most beautiful. According to the participant choices (aesthetic preferences within pairs), photos were ranked using the Elo algorithm^16,27^. More precisely, to correct for the effects of the order of matches in the final aesthetic scores of photos, we randomly simulated 1,000 orders of matches (Supplementary Fig. 1). We then computed the mean of the 1,000 bootstrapped final Elo scores of each photograph as the aesthetic score of each fishes.

### Effect of taxonomy on aesthetic scores

We performed an analysis of variance (ANOVA) to test the effect of each family on the mean aesthetic scores of each fishes (Supplementary Fig. 4).

### Effect of social background characteristics

Information on the social backgrounds of the observers was collected during the questionnaire to test for the effects of socio-professional factors and fish observation experiences on aesthetic preferences. These factors included gender, age, country of residence, occupation, professional category, qualifications and activity sector, and experience with diving, snorkelling, recreational or professional fishing and fishkeeping. Note that broadcasting the questionnaire via a website did not allow for control of the sampling of the observers (e.g. people living in France represented 64% of the sample). However, our objective was to control only for the effect of these factors, not to perform a detailed analysis of each factor. To this end, we performed ANOVA and tested the effect of each observer factor for each of the matches. We found a significant effect only for ‘diving’ (p-value = 0.009); however, this factor explained only a very small proportion of the variance (sum of squares = 0.004, F-value = 6.763). To better characterize the effect of diving on aesthetic preferences, preferences of non-divers have been compared to preferences of divers. To this end we recalculated the mean scores and standard deviation of each fishes by simulating 1,000 bootstrapped runs with randomly ordered matches using (i) matches judged by divers (n = 58,232 matches) and (ii) matches judged by non-divers (n = 98,124 matches). We then identified the significant differences in preferences (i.e. no overlap in the standard deviations of aesthetic scores, statistically confirmed with a Wilcoxon-Mann-Whitney test) for each fishes according to divers and non-divers.

### Functional space computation

To study the functional diversity of the fishes, we selected 6 categorical traits that describe coral fishes functional roles in aquatic ecosystems, mainly through regulation of food webs and nutrient cycling^17^ and that are available for a wide range of reef species (Supplementary Fig. 3): body size (common length), diet, mobility (sedentary; mobile within a reef; highly mobile i.e. between reefs), activity (period of the day during which fish are active: diurnal; diurnal & nocturnal; nocturnal), position (level in the water column: bottom; above bottom; pelagic), and schooling (gregariousness: solitary; pairing; small group; medium group; large group). We built a multidimensional functional space based on these traits values by computing a Principal Coordinate Analysis on the Gower’s distance between species and selected the 3 first axes^33^, which explained 87.62% of the variance. We performed ANOVAs to test the effect of each trait on the three axes of the functional space using p-values and the sum of squares as the percent of the explained variance (the sum of squares of each variable divided by the total sum of squares). PC1 was mainly explained by schooling (80%) and position (11%), whereas PC2 and PC3 were explained by mobility (60% and 30%) and activity (23% and 40%, Supplementary Fig. 3). The functional space obtained is represented in Fig. 2, and PC3 is not shown as it varies in the same way as PC2.

### Functional richness of more and less attractive species

We compared functional richness using the FRic index, which measures the volume occupied by a group of species within the functional space. Species with more extreme trait values will exhibit a higher FRic^18^. We sampled groups of fishes according to ascending and descending aesthetic scores, starting with the four most and least attractive fishes and expanding to all fishes, and calculated the FRic of each group. For each group size, we also computed the expected FRic by choosing fish randomly among the pool (1000 times) and calculated the mean expected FRic and standard deviation (Fig. 3a). We calculated the average difference between the most and least attractive fishes groups:

$$\frac{\sum _{i,j}(\frac{{F}_{i}-{F}_{j}}{{F}_{i}})\times 100}{n},$$

where *F*_*i*_ and *F*_*j*_ are the FRic values for the least and most attractive fishes groups, respectively (n groups).

### Data availability

The data that support the findings of this study (mean aesthetic scores, functional traits, coordinates in functional space) are available in figshare: 10.6084/m9.figshare.5151250.v1. The code used for the calculation of diversity indices is available at: http://villeger.sebastien.free.fr/Rscripts.html.

## Electronic supplementary material

Supplementary information
