# Supplementary material for: Confronting species aesthetics with ecological functions in coral reef fish
Source: Sci Rep. 2018 Aug 6;8:11733. doi: 10.1038/s41598-018-29637-7 (PMC6079033; doi:10.1038/s41598-018-29637-7)
Supplement: Supplementary file 1 — Supplementary information [file 41598_2018_29637_MOESM1_ESM.pdf]

Supplementary Figures

Confronting species aesthetics with ecological functions in coral reef fish

Anne-Sophie Tribot *[1], Quentin Carabeux[1], Julie Deter[2,3], Thomas Claverie[1,4], Sébastien Villéger[1], Nicolas Mouquet *[1]

1- MARBEC (MARine Biodiversity Exploitation and Conservation), Université de Montpellier, CNRS-IRD-IFREMER, Montpellier, France

2- Institut des Sciences de l'Evolution (ISEM)–UMR 5554 CNRS-UM-IRD, Université de Montpellier, Montpellier, France

3- Andromède Océanologie, Carnon, France

4- Centre Universitaire de formation et de recherche de Mayotte, Mayotte

E-mail addresses:

Quentin Carabeux: quentin.carabeux@orange.fr

Julie Deter: julie.deter@umontpellier.fr

Thomas Claverie: thomas.claverie@univ-mayotte.fr

Sébastien Villéger: sebastien.villeger@cnrs.fr

*Corresponding authors:

Anne-Sophie Tribot: anne-sophie.tribot@umontpellier.fr

Nicolas Mouquet: nicolas.mouquet@cnrs.fr

Supplementary Figure 1: Bootstrapping of the aesthetic scores

a) Variations in the aesthetic scores of three photos according to the number of matches played during the questionnaire, without bootstrapping. Top: aesthetic scores of the most attractive fish (*Pomacanthus semicirculatus*) calculated for 1,663 matches. Middle: aesthetic scores for the median fish (*Plectropomus laevis*) calculated for 1,706 matches. Below: aesthetic scores of the least attractive fish (*Labrichtys unilineatus* female) calculated for 1,652 matches. **b)** Stabilization of mean aesthetic scores during the bootstrap procedure (1,000-fold randomly ordered matches). Top: mean aesthetic score of the most attractive fish (*Pomacanthus semicirculatus*). Middle: mean aesthetic score of the median attractive fish (*Plectropomus laevis*). Below: mean aesthetic score of the least attractive fish (*Labrichtys unilineatus* female). Shaded areas correspond to standard deviations. Photographs: Randall, J. E. from FishBase.org.

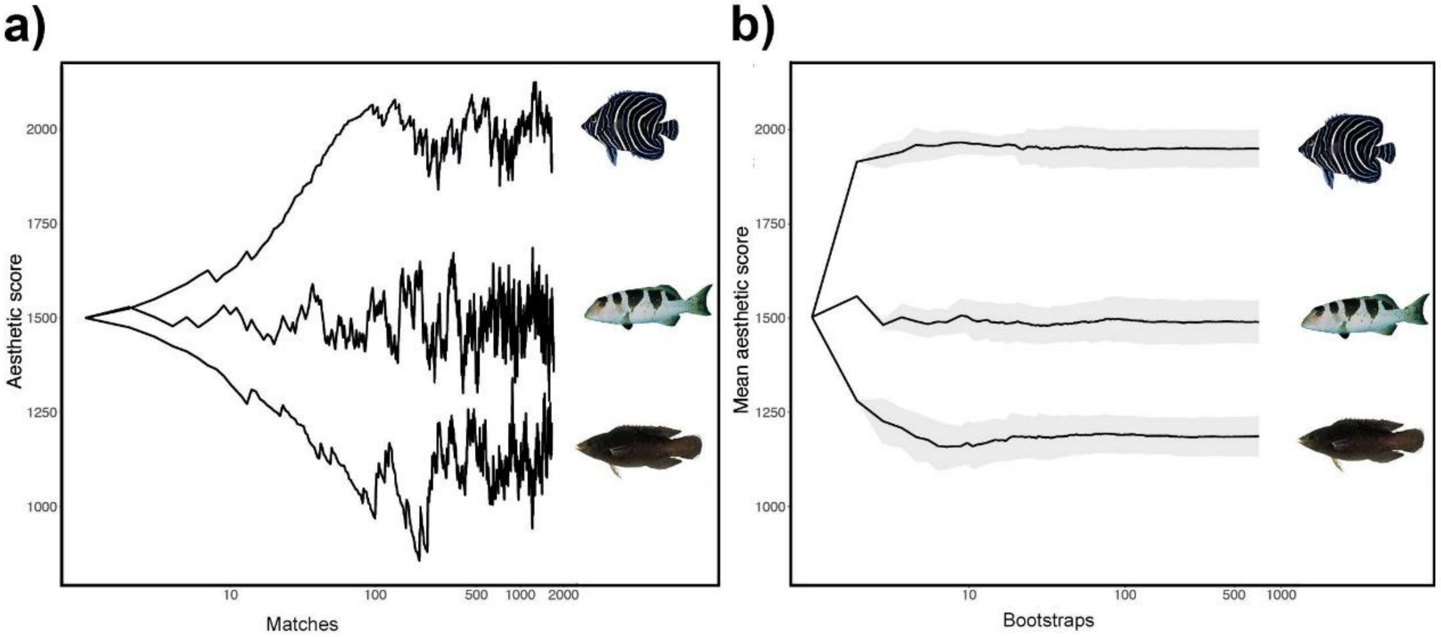

Supplementary Figure 2: Aesthetic scores according to divers and non-divers

Blue points: mean aesthetic scores calculated with matches judged by divers. Blue ribbons: standard deviations calculated with matches judged by divers. Green points: mean aesthetic scores calculated with matches judged by non-divers. Green ribbons: standard deviations calculated with matches judged by non-divers. Fish species shown: 60: *Chromis nigroanalis*, 102: *Monotaxis grandoculis* juvenile, 156: *Stethojulis albovittata* female, 86: *Kyphosus cinerascens*, 50: *Scarus niger* juvenile, 30: *Aulostomus chinensis*, 62: *Chromis viridis*, 123: *Parupeneus trifasciatus*, 19: *Arothron meleagris*, 109: *Naso brevirostris*, 79: *Gymnothorax favagineus*, 29: *Arothron stellatus*, 114: *Ostracion meleagris* male, 18: *Arothron meleagris*, 141: *Rhinecanthus aculeatus*, 116: *Oxymonacanthus longirostris*, 18, 19, 22, 23, 27, 28: *Arothron nigropunctatus*. For all species shown, differences between aesthetic scores for divers and non-divers were significant (Wilcoxon-Mann-Witney testn, all p-values $< 2x10^{-16}$). Photographs: Randall, J. E from FishBase.org.

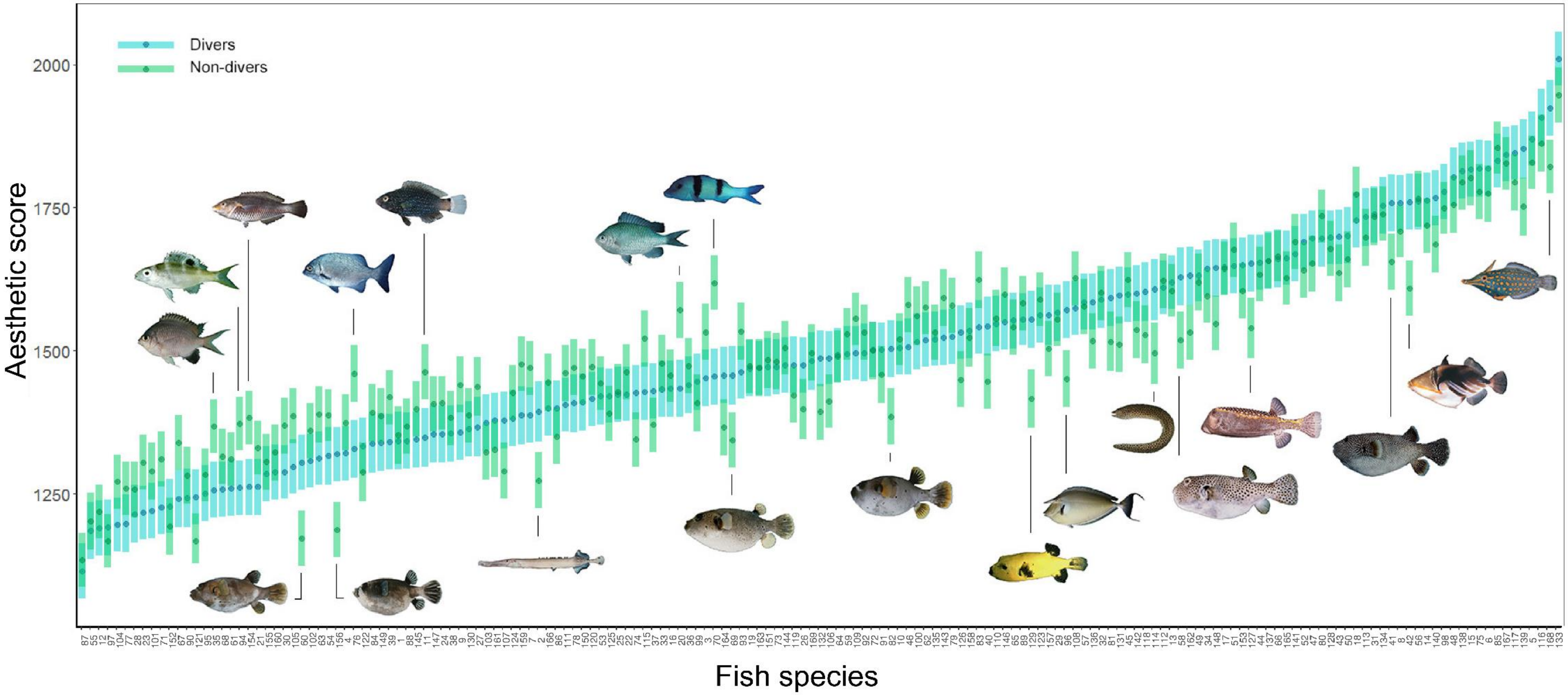

Supplementary Figure 3: Functional traits values along the three axes of the functional space.

PC1 explained 44.52% of the variance, PC2 explained 27.92%, and PC3 explained 15.16% (total = 87.62%). **Size**: 0-7 cm (S1); 7.1-15 cm (S2); 15.1- 30 cm (S3); 30.1-50 cm (S4); 50.1-80 cm (S5); >80 cm (S6). **Mobility**: sedentary (Sed); mobile within a reef (Mob); highly mobile i.e., between reefs (VMob). **Activity**: Diurnal; diurnal & nocturnal (Both); Nocturnal. **Schooling**: solitary (Sol); pairing (Pair); small group (SGroup); medium group (MGroup); large group (LGroup). **Position**: bottom (Bottom); above bottom (Low); pelagic (High). **Diet**: HD = herbivorous-detritivorous; HM = herbivorous macro-algal; IS = invertivorous sessile; IM = invertivorous mobile; PK = planktonivorous; FC = Pelagic macro-organisms; OM = omnivorous (herbivorous and/or detritivorous AND carnivorous). **Sq**: Sum of squares of each variable calculated using ANOVA divided by the total sum of squares.

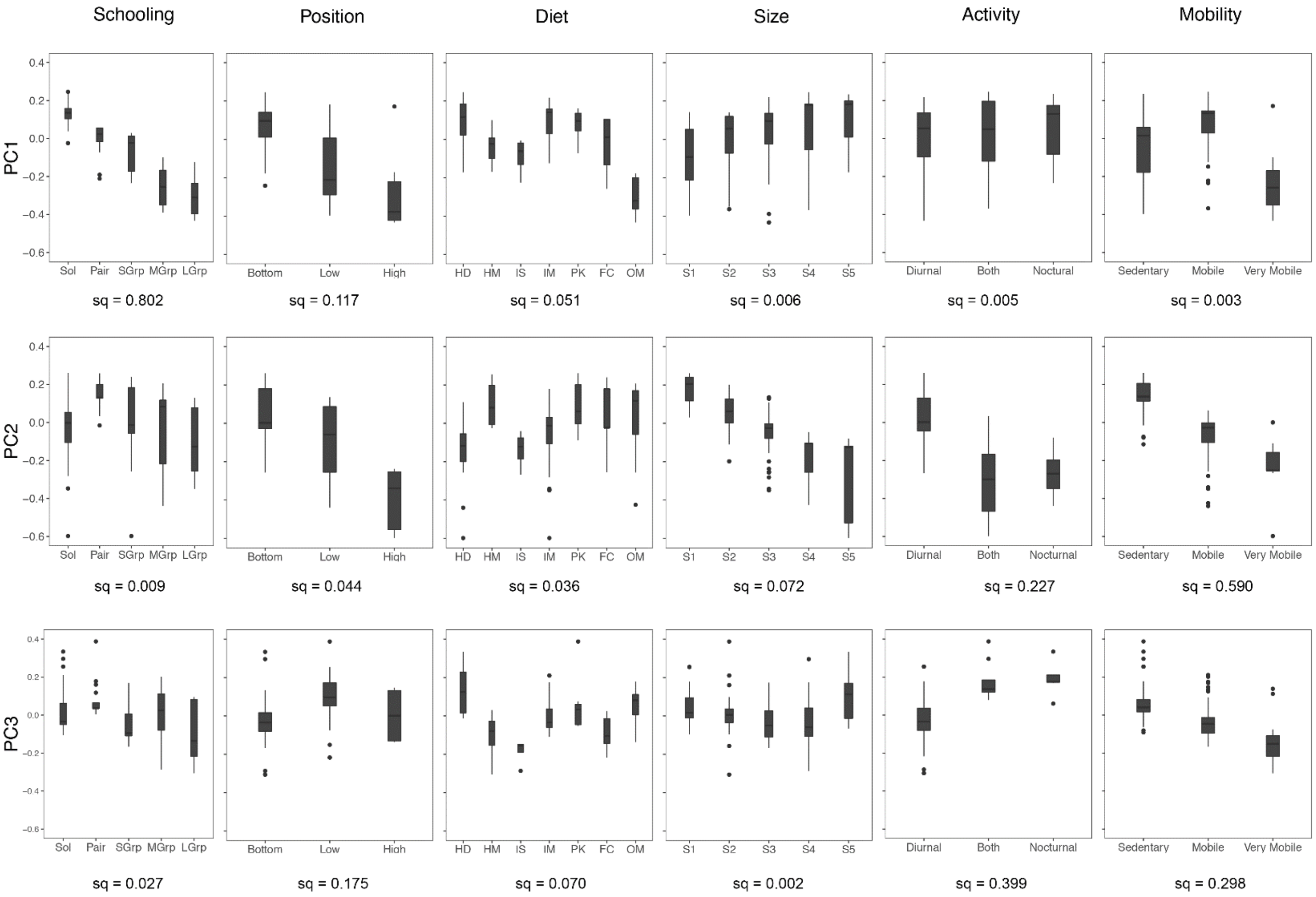

Supplementary Figure 4: Mean aesthetic scores of families

Mean and standard deviations of aesthetic scores for each family. P-value of variable 'Family' in the model = 6.927×10^{-4}, sum of squares = 0.324. The asterisks represent the p-values of an ANOVA that tests the effect of each family on the mean aesthetic scores of fish: '***' < 0.001, '*' < 0.05. For Zanclidae, the sum of squares (sum of squares of the family divided by total sum of squares) = 0.028, Scorpaenidae = 0.020, Monacanthidae = 0.022, Chaetodontidae = 0.091, Lutjanidae = 0.021.

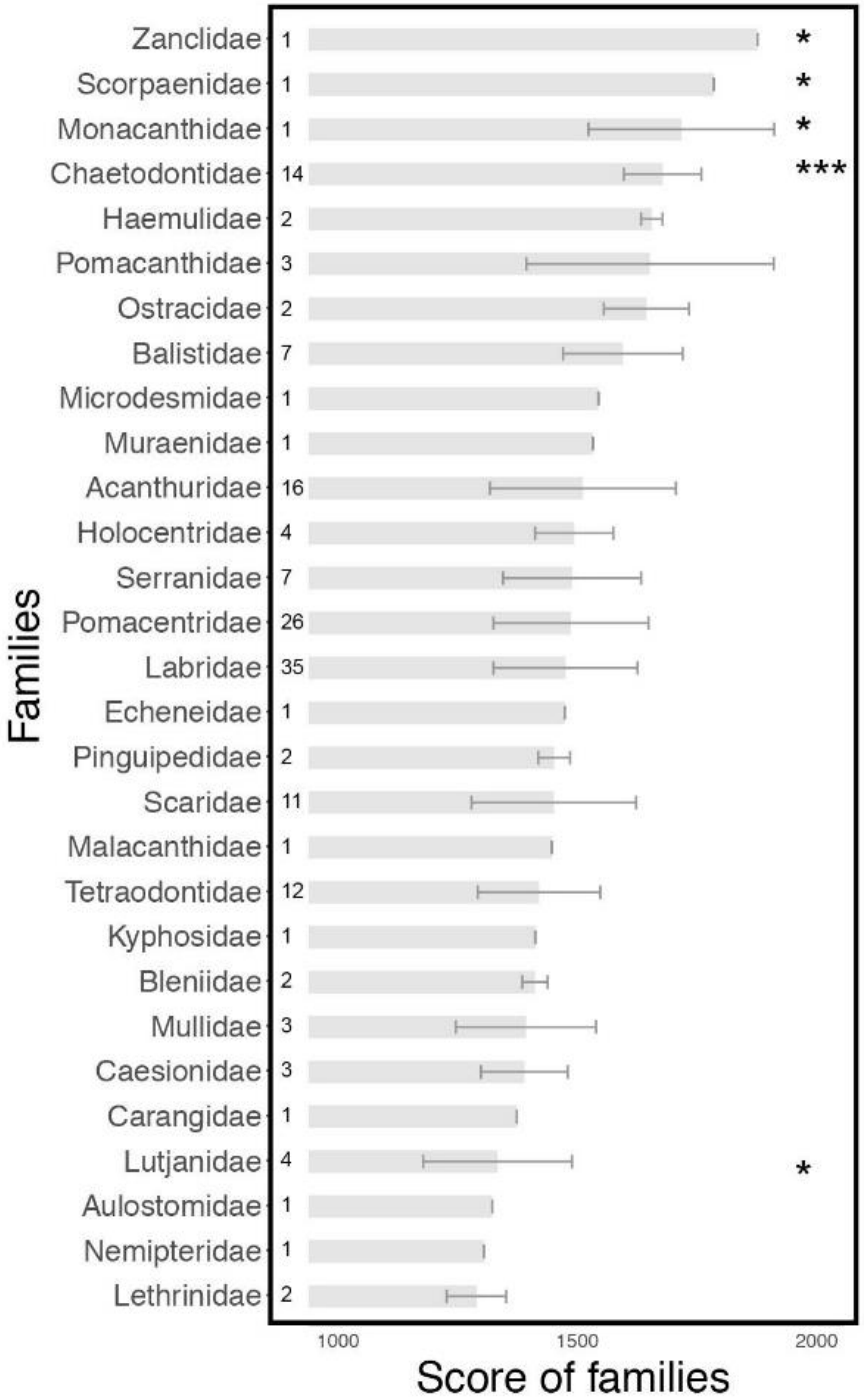

Supplementary Table 1: List of fish species

Species name	Species number	Mean Aesthetic score	SD Aesthetic score	Family	Genus
Abudefduf_notatus	1	1362.977	47.051	Pomacentridae	Abudefduf
Abudefduf_sparoides	2	1408.883	48.377	Pomacentridae	Abudefduf
Abudefduf_viagiensis	3	1447.977	50.552	Pomacentridae	Abudefduf
Acanthrurs_nigricauda	4	1345.342	49.401	Acanthuridae	Acanthurus
Acanthurus_leucosternon	5	1843.976	47.021	Acanthuridae	Acanthurus
Acanthurus_lineatus	6	1800.780	48.461	Acanthuridae	Acanthurus
Acanthurus_nigrofuscus	7	1405.228	47.845	Acanthuridae	Acanthurus
Acanthurus_tennenti	8	1730.225	48.758	Acanthuridae	Acanthurus
Acanthurus_thompsoni	9	1379.417	48.272	Acanthuridae	Acanthurus
Acanthurus_triostegus	10	1517.963	47.299	Acanthuridae	Acanthurus
Acanthurus_tristis	11	1371.510	48.075	Acanthuridae	Acanthurus
Aethaloperca_rogaa	12	1199.715	48.532	Serranidae	Aethaloperca
amphiprion_akallopisos	13	1597.343	49.716	Pomacentridae	Amphiprion
amphiprion_latifasciatus	14	1754.417	47.681	Pomacentridae	Amphiprion
Anampses_meleagrides_fem	15	1787.457	49.224	Labridae	Anampses
Anampses_meleagrides_male	16	1441.156	49.453	Labridae	Anampses
Anampses_twistii	17	1617.757	46.994	Labridae	Anampses
Arothron_meleagris_black	18	1688.140	49.902	Tetraodontidae	Arothron
Arothron_meleagris_yellow	19	1467.979	49.724	Tetraodontidae	Arothron
Arothron_nigropunctatus1	20	1443.158	49.406	Tetraodontidae	Arothron
Arothron_nigropunctatus2	21	1305.013	48.946	Tetraodontidae	Arothron
Arothron_nigropunctatus3	22	1433.405	49.662	Tetraodontidae	Arothron
Arothron_nigropunctatus4	23	1243.354	47.143	Tetraodontidae	Arothron
Arothron_nigropunctatus5	24	1372.886	47.193	Tetraodontidae	Arothron
Arothron_nigropunctatus6	25	1431.619	48.677	Tetraodontidae	Arothron
Arothron_nigropunctatus7	26	1478.849	51.280	Tetraodontidae	Arothron
Arothron_nigropunctatus8	27	1385.286	51.214	Tetraodontidae	Arothron
Arothron_nigropunctatus9	28	1231.306	47.802	Tetraodontidae	Arothron
Arothron_stellatus	29	1556.810	50.661	Tetraodontidae	Arothron
Aulostolus_chinensis	30	1321.839	52.720	Aulostomidae	Aulostomus
Balistapus_undulatus	31	1712.549	48.781	Balistidae	Balistapus
Balistoides_viridescens	32	1566.473	50.464	Balistidae	Balistoides
Bodianus_axillaris	33	1439.169	47.090	Lanridae	Bodianus
Bodianus_axillaris_juv	34	1614.913	47.600	Lanridae	Bodianus
Caesio_lunaris	35	1284.753	46.453	Caesionidae	Caesio
Caesio_suevica	36	1446.285	50.000	Caesionidae	Caesio
Caesio_xanthonota	37	1437.194	47.910	Caesionidae	Caesio
Caranx_melampygus	38	1373.528	48.708	Carangidae	Caranx
Centropyge_multispinis	39	1356.901	49.226	Pomacanthidae	Centropyge
Cephalopholis_argus	40	1543.276	48.568	Serranidae	Cephalopholis

Chaetodon_auriga	41	1726.262	48.788	Chaetodontidae	Chaetodon
Chaetodon_auriga_juv	42	1731.945	47.599	Chaetodontidae	Chaetodon
Chaetodon_falcula	43	1682.616	46.980	Chaetodontidae	Chaetodon
Chaetodon_guttatissimus	44	1641.312	47.680	Chaetodontidae	Chaetodon
Chaetodon_kleinii	45	1579.544	47.697	Chaetodontidae	Chaetodon
Chaetodon_kleinii2	46	1521.509	50.357	Chaetodontidae	Chaetodon
Chaetodon_melannotus	47	1665.913	48.025	Chaetodontidae	Chaetodon
Chaetodon_meyeri	48	1771.345	50.962	Chaetodontidae	Chaetodon
Chaetodon_trifascialis	49	1610.986	47.149	Chaetodontidae	Chaetodon
Chaetodon_trifasciatus	50	1683.970	48.162	Chaetodontidae	Chaetodon
Chaetodon_vagabundus	51	1621.902	45.466	Chaetodontidae	Chaetodon
Chateodon_lunula	52	1665.822	50.258	Chaetodontidae	Chaetodon
Cheilinus_fasciatus	53	1428.620	48.515	Labridae	Cheilinus
Cheilinus_fasciatus_juv	54	1343.000	46.447	Labridae	Cheilinus
Chlorurus_sordidus_fem	55	1176.764	48.792	Scaridae	Chlorurus
Chlorurus_sordidus_male	56	1742.657	49.121	Scaridae	Chlorurus
choerodon_graphicus	57	1562.662	49.215	Labridae	Choerodon
Chromis_atripectoralis	58	1607.506	47.546	Pomacentridae	Chromis
Chromis_dimidiata	59	1493.152	50.259	Pomacentridae	Chromis
Chromis_nigroanalis	60	1329.235	48.689	Pomacentridae	Chromis
Chromis_ternatensis	61	1297.676	48.549	Pomacentridae	Chromis
Chromis_viridis	62	1523.562	50.016	Pomacentridae	Chromis
Chromis_weberi	63	1341.033	46.362	Pomacentridae	Chromis
Chrysiptera_annulata	64	1492.838	48.422	Pomacentridae	Chrysiptera
Coris_aygula_fem	65	1550.794	51.033	Labridae	Coris
Coris_aygula_juv	66	1658.440	47.517	Labridae	Coris
Coris_aygula_male	67	1264.187	48.419	Labridae	Coris
Ctenochaetus_striatus	68	1295.176	48.882	Pomacentridae	Chrysiptera
Dascyllus_aruanus	69	1457.546	49.744	Pomacentridae	Dascyllus
Dascyllus_carneus	70	1449.161	47.452	Pomacentridae	Dascyllus
Dascyllus_trimaculatus	71	1246.354	47.128	Pomacentridae	Dascyllus
Dascyllus_trimaculatus_juv	72	1502.770	50.198	Pomacentridae	Dascyllus
Echeneis_naucrates	73	1473.537	48.099	Echeneidae	Echeneis
Epinephelus_areolatus	74	1436.011	49.744	Serranidae	Epinephelus
Forcipiger_longirostris	75	1787.611	48.294	Chaetodontidae	Forcipiger
Gomphosus_caeruleus_femelle	76	1346.585	49.318	Labridae	Gomphosus
Gomphosus_caeruleus_juv	77	1211.942	48.033	Labridae	Gomphosus
Gomphosus_caeruleus_male	78	1418.441	49.103	Labridae	Gomphosus
Gymnothorax_favagineus	79	1532.579	53.479	Muraenidae	Gymnothorax
Halichoeres_cosmetus_fem	80	1666.229	48.332	Labridae	Halichoeres
Halichoeres_cosmetus_male	81	1572.430	45.261	Labridae	Halichoeres
Halichoeres_hortulanus_female	82	1514.069	47.062	Labridae	Halichoeres
Halichoeres_hortulanus_male	83	1543.035	48.728	Labridae	Halichoeres
Hemigymnus_fasciatus	84	1356.386	47.818	Labridae	Hemigymnus
Heniochus_acuminatus	85	1803.594	47.824	Chaetodontidae	Heniochus
Kyphosus_cinerascens	86	1412.319	50.225	Kyphosidae	Kyphosus
Labrichtys_unilineatus_fem	87	1128.443	48.766	Labridae	Labrichtys
Labrichtys_unilineatus_juv	88	1365.743	50.577	Labridae	Labrichtys

Labrichtys_unilineatus_male	89	1551.732	48.594	Labridae	Labrichtys
Labroides_bicolor	90	1269.355	51.166	Labridae	Labroides
Labroides_dimidiatus	91	1508.537	46.383	Labridae	Labroides
Labropsis_xanthonota	92	1501.658	48.338	Labridae	Labroides
Labropsis_xanthonota_juv	93	1463.703	48.383	Labridae	Labropsis
Lutjanus_bohar	94	1298.740	48.167	Lutjanidae	Lutjanus
Lutjanus_fulviflamma	95	1276.153	46.797	Lutjanidae	Lutjanus
Lutjanus_kasmira	96	1557.753	47.936	Lutjanidae	Lutjanus
Macolor_niger	97	1199.943	49.327	Lutjanidae	Macolor
Macolor_niger_juv	98	1761.500	48.884	Balistidae	Macolor
Malacanthus_lattovitatus	99	1446.719	48.874	Malacanthidae	Malacanthus
Melichthys_niger	100	1523.516	52.405	Balistidae	Melichthys
Monotaxis_grandoculis	101	1245.255	50.108	Lethrinidae	Monotaxis
Monotaxis_grandoculis_juv	102	1333.121	47.401	Lethrinidae	Monotaxis
Myripristis_violacea	103	1389.331	49.087	Holocentridae	Myripristis
Naso_brachycentron	104	1207.083	47.555	Acanthuridae	Naso
Naso_brevirostris_fem	105	1327.240	49.903	Acanthuridae	Naso
Naso_brevirostris_juv	106	1489.458	50.558	Acanthuridae	Naso
Naso_brevirostris_male	107	1391.025	48.615	Acanthuridae	Naso
Naso_elegans	108	1558.736	49.272	Acanthuridae	Naso
Naso_unicornis	109	1497.666	50.266	Acanthuridae	Naso
Nemateleotris_magnifica	110	1544.072	49.357	Microdesmidae	Nemateleotris
Neoglyphidodon_melas	111	1412.636	52.149	Pomacentridae	Neoglyphidodon
Neoglyphidodon_melas_juv	112	1592.078	46.828	Pomacentridae	Neoglyphidodon
Ostracion_meleagris_fem	113	1707.288	50.135	Ostracidae	Ostracion
Ostracion_meleagris_male	114	1581.139	49.516	Ostracidae	Ostracion
Oxycheilinus_digramma	115	1436.700	46.121	Labridae	Oxycheilinus
Oxymonacanthus_longirostris	116	1854.461	48.428	Monacanthidae	Oxymonacanthus
Paracanthurus_hepatus	117	1832.893	47.503	Acanthuridae	Paracanthurus
Paraluteres_prionurus	118	1579.873	49.883	Monacanthidae	Paraluteres
Parapercis_hexophtalma_fem	119	1474.912	46.440	Pinguipedidae	Parapercis
Parapercis_hexophtalma_male	120	1427.876	47.987	Pinguipedidae	Parapercis
Parupeneus_cyclostomus	121	1271.789	49.161	Mullidae	Parupeneus
Parupeneus_macronema	122	1350.852	50.593	Mullidae	Parupeneus
Parupeneus_trifasciatus	123	1555.653	48.858	Mullidae	Parupeneus
Plagiotremus_rhinorhynchos_black	124	1392.489	49.938	Bleniidae	Plagiotremus
Plagiotremus_rhinorhynchos_orange	125	1430.339	48.667	Bleniidae	Plagiotremus
Plectopromus_pessuliferus	126	1535.095	47.039	Serranidae	Plectropomus
Plectorhinchus_vittatus	127	1639.710	48.159	Haemulidae	Plectorhinchus
Plectorhinchus_vittatus_juv	128	1671.393	48.218	Haemulidae	Plectorhinchus
Plectroglyphidodon_dickii	129	1554.100	48.914	Pomacentridae	Plectroglyphidodon
Plectroglyphidodon_johnstonianus	130	1384.192	48.808	Pomacentridae	Plectroglyphidodon
Plectroglyphidodon_lacrymatus	131	1578.811	47.948	Pomacentridae	Plectroglyphidodon
Plectropomus_laevis	132	1489.358	47.171	Serranidae	Plectropomus
Pomacanthus_semicirculatus	133	1964.861	46.982	Pomacentridae	Pomacentrus
Pomacentrus_caeruleus	134	1719.621	49.891	Pomacentridae	Pomacentrus
Pomacentrus_sulfureus	135	1529.585	47.039	Pomacentridae	Pomacentrus
Pseudanthias_squamipinnis_fem	136	1566.014	48.306	Serranidae	Pseudanthias

Pseudanthias_squamipinnis_male	137	1653.831	50.897	Serranidae	Pseudanthias
Pterois_volitans	138	1784.876	52.496	Scorpaenidae	Pterois
Pygoplites_diacanthus	139	1839.414	47.570	Pomacanthidae	Pygoplites
Pygoplites_diacanthus_juv	140	1758.483	48.316	Pomacanthidae	Pygoplites
Rhinecanthus_aculeatus	141	1661.760	49.398	Balistidae	Rhinecanthus
Sargocentron_caudimaculatus	142	1579.747	49.011	Holocentridae	Sargocentron
Sargocentron_diadema	143	1530.232	48.613	Holocentridae	Sargocentron
Sargocentron_praslin	144	1473.850	50.619	Holocentridae	Sargocentron
Scarus_caudofasciatus_fem	145	1368.838	50.551	Scaridae	Scarus
Scarus_caudofasciatus_male	146	1545.727	49.015	Scaridae	Scarus
Scarus_frenatus_fem	147	1372.558	50.968	Scaridae	Scarus
Scarus_frenatus_male	148	1616.512	48.294	Scaridae	Scarus
Scarus_niger_fem	149	1356.840	47.290	Scaridae	Scarus
Scarus_niger_juv	150	1420.512	51.393	Scaridae	Scarus
Scarus_niger_male	151	1471.099	48.460	Scaridae	Scarus
Scarus_tricolor_fem	152	1247.431	49.961	Scaridae	Scarus
Scarus_tricolor_male	153	1635.935	47.338	Scaridae	Scarus
Scolopsis_ghanam	154	1304.879	48.829	Nemipteridae	Scolopsis
Stegastes_pelicieri	155	1307.369	48.313	Pomacentridae	Stegastes
stethojulis_albovittata_fem	156	1343.595	48.992	Labridae	Stethojulis
stethojulis_albovittata_male	157	1556.294	46.996	Labridae	Stethojulis
sufflamen_bursa	158	1539.378	49.673	Balistidae	Sufflamen
Sufflamen_chrysopterum	159	1398.014	48.922	Balistidae	Sufflamen
Thalassoma_amblycephalum_fem	160	1307.490	49.536	Labridae	Thalassoma
Thalassoma_amblycephalum_male	161	1390.949	48.117	Labridae	Thalassoma
Thalassoma_hardwicke	162	1609.134	47.277	Labridae	Thalassoma
Thalassoma_hebraicum	163	1470.280	47.883	Labridae	Thalassoma
Thalassoma_lunare_fem	164	1456.690	47.696	Labridae	Thalassoma
Thalassoma_lunare_male	165	1658.945	48.012	Labridae	Thalassoma
Thalassoma_purpureum_fem	166	1410.546	48.752	Labridae	Thalassoma
Thalassoma_purpureum_male	167	1809.357	47.284	Labridae	Thalassoma
Zanclus_cornutus	168	1876.378	49.681	Zanclidae	Zanclus
Zebrasoma_scopas	169	1482.712	46.801	Acanthuridae	Zebrasoma
